# A RNA Interference Screen Identifies the Protein Phosphatase 2A Subunit PR55γ as a Stress-Sensitive Inhibitor of c-SRC

**DOI:** 10.1371/journal.pgen.0030218

**Published:** 2007-12-07

**Authors:** Pieter J. A Eichhorn, Menno P Creyghton, Kevin Wilhelmsen, Hans van Dam, René Bernards

**Affiliations:** 1 Division of Molecular Carcinogenesis, The Netherlands Cancer Institute, Amsterdam, The Netherlands; 2 Division of Cell Biology, The Netherlands Cancer Institute, Amsterdam, The Netherlands; 3 Center for Biomedical Genetics, The Netherlands Cancer Institute, Amsterdam, The Netherlands; 4 Department of Molecular Cell Biology, Leiden University Medical Center, Leiden, The Netherlands; Fred Hutchinson Cancer Research Center, United States of America

## Abstract

Protein Phosphatase type 2A (PP2A) represents a family of holoenzyme complexes with diverse biological activities. Specific holoenzyme complexes are thought to be deregulated during oncogenic transformation and oncogene-induced signaling. Since most studies on the role of this phosphatase family have relied on the use of generic PP2A inhibitors, the contribution of individual PP2A holoenzyme complexes in PP2A-controlled signaling pathways is largely unclear. To gain insight into this, we have constructed a set of shRNA vectors targeting the individual PP2A regulatory subunits for suppression by RNA interference. Here, we identify PR55γ and PR55δ as inhibitors of c-Jun NH_2_-terminal kinase (JNK) activation by UV irradiation. We show that PR55γ binds c-SRC and modulates the phosphorylation of serine 12 of c-SRC, a residue we demonstrate to be required for JNK activation by c-SRC. We also find that the physical interaction between PR55γ and c-SRC is sensitive to UV irradiation. Our data reveal a novel mechanism of c-SRC regulation whereby in response to stress c-SRC activity is regulated, at least in part, through loss of the interaction with its inhibitor, PR55γ.

## Author Summary

Protein Phosphatase type 2A (PP2A) represent a family of holoenzyme complexes involved in wide range of activities such as growth, differentiation, and cell death. The PP2A holoenzyme complex is made up of a catalytic, a structural, and one of various “B” subunits. These “B” subunits are thought to provide the substrate specificity required for PP2A activity. Previous work on PP2A has mostly been derived by inhibiting the catalytic subunit through chemical inhibition, as such inhibiting all of the pathways associated with PP2A. To identify individual “B” subunits involved in specific cellular processes we have generated a “B” subunit gene knockdown library, which allows us to inhibit each of the known “B” subunits individually. One of the many pathways regulated by PP2A is the c-Jun NH_2_-terminal kinase (JNK) kinase pathway, which, depending on stimulus, can affect either cell survival or cell proliferation. Here we report that the “B” subunit PR55γ acts as a negative regulator of JNK activity and cell death. We show that PR55γ influences JNK activity by inhibiting one of its upstream regulators, the proto-oncogene c-SRC, through dephosphorylation at one of the key residues on c-SRC, a site we show to be critical for c-SRC activation following cell stress. Overall our work describes the novel function of a specific PP2A subunit involved in cell survival and identifies a novel mechanism of c-SRC regulation.

## Introduction

The Src family of nonreceptor tyrosine kinases are integral players in the mediation of various physiological processes such as cell motility, adhesion, proliferation, and survival [1]. Members of the Src family share a conserved structure consisting of four Src homology (SH) domains, a unique region, and a short negative regulatory tail. The amino terminal SH4 domain is myristoylated and targets the protein to the membrane, while the carboxy-terminal SH1 domain functions as a tyrosine kinase domain [2]. c-SRC activation is negatively regulated by Carboxy Src Kinase (CSK) or its homologue CHK through Tyrosine 527 (Tyr^527^) phosphorylation [2]. This inhibitory phosphorylation promotes the assembly of the SH2, SH3, and kinase domains into a closed conformation [2]. Following stimulation by various stresses and growth factors c-SRC activation is initiated by dephosphorylation of the Tyr^527^ residue by the protein-tyrosine phosphatase PTPα [3] and PTP1B [4]. Alternatively, c-SRC is activated by the binding of tyrosine-phosphorylated proteins to the SH2 domain, resulting in destabilization of the intermolecular interaction between Tyr^527^ and the SH2 domain [2]. Subsequently, c-SRC is autophosphorylated at Tyrosine 416 (Tyr^416^), a site within a segment of the kinase domain termed the activation loop, promoting a conformational change that allows the kinase to adopt an open active confirmation [2].

c-SRC is overexpressed or activated in a wide variety of tumors [5,6]. However, overexpression of c-SRC by itself has only minor oncogenic potential [7] and mutations in c-SRC in cancer have only been found sporadically [8]. This led to the hypothesis that c-SRC has a supportive function in tumorigenesis rather than a role in the actual transformation process [9]. Overexpression of v-Src, a constitutively active form of c-SRC lacking the c-terminal part containing the inhibitory Tyr^527^, is a potent activator of c-Jun NH_2_-terminal kinase (JNK), a growth-regulatory enzyme that can control cell proliferation and cell survival both positively and negatively, depending on the stimulus and the cellular context [10,11]. Furthermore, SRC activity is essential for JNK activation following a number of different stress stimuli, including UV irradiation [12–14].

Protein Phosphatase 2A (PP2A) is a serine/threonine phosphatase that can influence the phosphorylation state of many signaling enzymes [15,16], and inhibition of this phosphatase can affect cellular responses such as growth, differentiation, and apoptosis [15,17]. The holoenzyme generally exists as a core dimer, consisting of a 36-kDa catalytic subunit (PP2Ac) and a 65-kDa scaffold subunit (PR65) that associates with a variety of regulatory subunits. These regulatory B subunits can modulate the activity of the PP2Ac/PR65 core unit, thus allowing specific temporal targeting of a wide range of PP2A substrates. To date 15 genes coding for more than 26 (B) regulatory subunits have been identified that are subdivided into five different subfamilies [17].

The variable PP2A B subunits are targeted by a number of viral oncogenes, which thereby compete for interaction with the PR65/PP2Ac core dimer. This suggests that specific PP2A holoenzymes play a role in viral propagation and oncogenic transformation [18,19], which is further supported by the finding that general inhibitors of PP2A can cause tumor growth on the skin and liver of rodents [20–23]. Understanding the precise manner in which PP2A is involved in the regulation of these different signaling cascades and its role during oncogenic transformation requires the identification of the specific holoenzymes involved in these processes. Interpretation of a large amount of data using general PP2A inhibitors has been limited by the pleiotropic inhibition of all PP2A holoenzyme complexes by the inhibitors used. Furthermore, ectopic expression of the various B subunits can lead to competition with other subunits for binding to the holoenzyme, making it difficult to draw firm conclusions from the data [24,25]. Using a gene family knockdown library targeting all deubiquitinating (DUB) enzymes, we previously identified the familial tumor suppressor gene *CYLD* as a novel regulator of the NF-κB signaling pathway [26] and USP1 as the deubiquitinating enzyme of the FANCD2 DNA repair protein [27]. To study the role of the various PP2A complexes in specific pathways we have constructed a library of 61 independent vectors expressing short hairpin RNAs (shRNA) targeting the PP2A regulatory B subunits for suppression. Using this knockdown library in a screen for enhancers of JNK activation following cellular stress, we identified a number of PP2A B subunits as novel regulators of JNK activation, most notably PR55γ and PR55δ. Furthermore we demonstrate that the PP2A B subunit PR55γ negative regulates the JNK effector pathway by acting as a stress sensitive inhibitor of c-SRC activity.

## Results

### PR55γ Is a Modulator of JNK Activity

**Figure 1:**
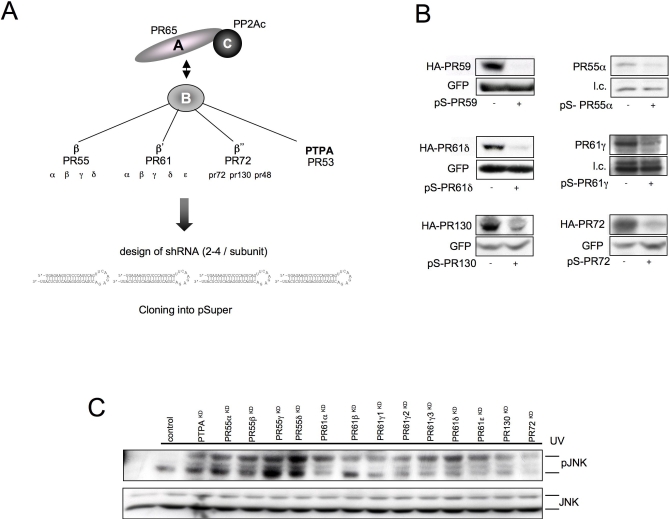
PP2A Family Screen (A) Schematic of the PP2A holoenzyme and outline of the B regulatory subunit families. (B) U2-OS cells were transfected with the indicated pSuper constructs and where available cotransfected with an HA-tagged version of the corresponding PP2A B subunit. Immunoblot panels show the efficiency of knockdown in six different pools as judged by the ability to knockdown cotransfected or endogenous protein (GFP is a transfection control). (C) U2-OS cells were cotransfected with pooled PP2A shRNAs or a control vector. Levels of phosphorylated JNK (α-pJNK) or JNK1 and JNK2 (α-JNK) were shown in cell lysates for the different samples 60 min after UV treatment of the cells. doi:10.1371/journal.pgen.0030218.g001

To identify the specific PP2A holoenzyme complexes involved in pathways known to be modulated by PP2A, we constructed a gene family knockdown library targeting all putative human PP2A regulatory B subunits for suppression. We retrieved the cDNA sequences for each of the PP2A subunit family members from the ENSEMBL database and designed two to four unique 19-mer sequences for each transcript for cloning into pSuper and pRetro-Super [26,28]. In total 61 knockdown vectors were generated, which were then subsequently pooled into 16 sets of two to four vectors per transcript with each set targeting one of the regulatory B subunits or a specific transcript variant (Figure 1A; Table S1). To validate the pooled knockdown vectors, we tested six randomly chosen pools of vectors for their ability to effectively knockdown the target proteins. All pools tested show a notable reduction in target protein expression levels (Figure 1B).

**Figure 2:**
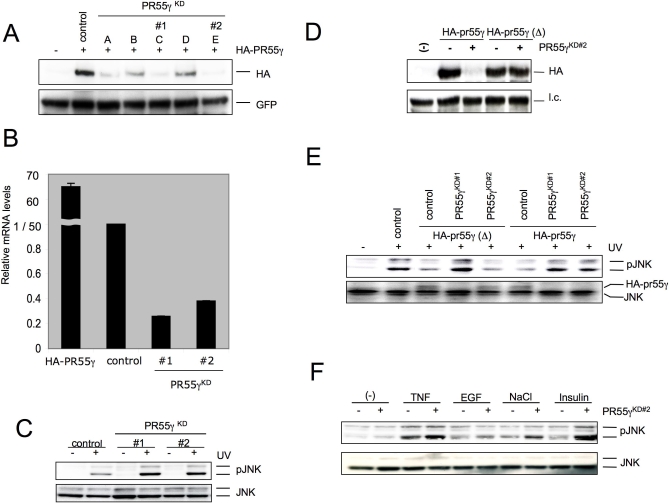
PR55γ Is a Regulator of JNK following UV Irradiation (A) U2-OS cells expressing pcDNA-HA-PR55γ were cotransfected with the pooled knockdown (PR55γ^KD^) vectors as indicated (A–E) or a control vector. GFP expression serves as a measure of transfection consistency. (B) U2-OS cells were cotransfected with PR55γ^KD^ vectors #1 or #2, pcDNA-PR55γ serves as a positive control. pSuper vector targeting a mouse PP2A subunit PR59 served as an shRNA control. mRNA levels relative to the control are shown as evaluated by quantitative real-time PCR. (C) U2-OS cells were cotransfected with PR55γ^KD^ vectors as indicated (#1 or #2) or control vector. Selected cells were exposed to UV irradiation (100 J/m^2^) and incubated for a further 60 min. Protein samples were analyzed by immunoblotting with antibodies targeting phosphorylated JNK (α-pJNK) or JNK1 and JNK2 (α-JNK). (D) U2-OS cells expressing pcDNA-HA-PR55γ or pcDNA-HA-PR55γ (Δ) were cotransfected with PR55γ^KD^ vector #2. Protein samples were analyzed by immunoblotting with antibodies targeting HA. (E) U2-OS cells expressing pcDNA-HA-PR55γ, pcDNA-HA- PR55γ(Δ), or a control vector were cotransfected with PR55γ^KD^ vectors #1 or #2. A pSuper vector targeting a mouse PP2A subunit PR59 served as an shRNA control. Selected cells were exposed to UV irradiation (100 J/m^2^) and incubated for a further 60 min. Protein samples were analyzed by immunoblotting with antibodies targeting phosphorylated JNK (α- pJNK), total JNK (α-JNK), or haemoglutinin (α-HA, reprobe). (F) U2-OS cells expressing PR55γ^KD2^ vector or a control vector exposed to TNF-α, EGF, NaCl, or insulin for 5 min and incubated for a further 30–60 minutes. pJNK relative to total JNK levels are shown. doi:10.1371/journal.pgen.0030218.g002

Studies using viral proteins that target the regulatory B subunits of the PP2A holoenzyme complex indicate that JNK and the proto-oncogene c-Jun can be regulated by PP2A [29]. This suggests that specific PP2A regulatory B subunits are involved in PP2A-mediated regulation of the JNK pathway. To directly assess the putative role of PP2A in JNK regulation we asked if suppression of one or more PP2A regulatory subunits by RNA interference could affect JNK activity following UV irradiation. U2-OS cells were transfected with the different library pools and then assayed by western blotting for the efficiency of UV induced JNK activation as judged by threonine-183/tyrosine-185 phosphorylation. Unsurprisingly, we found that suppression of a number of the B subunits appeared to enhance the levels of phosphorylated JNK following UV. Of these, PR55γ consistently yielded the strongest effect and was chosen for further validation (Figure 1C and unpublished data). To evaluate which of the four individual knockdown vectors in this pool were active against PR55γ, we transfected cells with HA-tagged PR55γ and determined the protein levels of HA-PR55γ in lysates of transfected cells in the presence or absence of the individual PR55γ knockdown vectors. As depicted in Figure 2A, all four shRNA vectors (A–D) in this pool were able to suppress HA-PR55γ expression levels, whereas no effect was detected on a cotransfected green fluorescent protein (GFP) (Figure 2A). A shRNA targeting the mouse-specific B subunit PR59 was used as a negative control in all experiments. Vectors A and C were more efficient in suppressing HA-PR55γ protein levels than vectors B and D (Figure 2A). A fifth knockdown vector (E) was designed, which like vector C, induced strong suppression of ectopic PR55γ expression (Figure 2A). shRNAs C and E will be referred from here on as shRNA#1 and shRNA#2, respectively.

To test whether these shRNAs #1 and #2 could inhibit endogenous PR55γ levels we performed quantitative real-time PCR (QRT-PCR). We found that both shRNAs efficiently suppressed endogenous PR55γ mRNA levels (Figure 2B). Furthermore, inhibition of PR55γ with both validated knockdown vectors could efficiently enhance the activation of JNK by UV irradiation (Figure 2C), arguing against an off-target effect of the shRNAs. This result underscores the validity of the screen and suggests that endogenous PR55γ is a repressor of stress-induced JNK activation.

To determine whether the activation of JNK after transfection of PR55γ knockdown vectors was a consequence of the loss of PR55γ expression, we performed an add-back experiment. To do this we restored PR55γ levels to the control situation using a PR55γ construct (ΔPR55γ) containing two noncoding mutations within the region targeted by knockdown vector #2, rendering it refractory to shRNA-mediated suppression (Figure 2D). We found that expression of ΔPR55γ completely abolished the enhanced activation of UV-induced JNK observed with shRNA vector #2, but not with shRNA vector #1, which targets a region that was not mutated in ΔPR55γ (Figure 2E). These results argue that the effects of the knockdown vectors targeting PR55γ for shRNA-mediated suppression on JNK activation are the result of loss of PR55γ.

To investigate whether the enhanced JNK activation upon PR55γ knockdown is specific for UV irradiation, we asked whether other stimuli that lead to the activation of the JNK pathway might also be enhanced by loss of PR55γ. We found that TNFα, insulin, and osmotic stress-mediated JNK activation could all be enhanced by suppression of PR55γ but not EGF-mediated JNK activation (Figure 2F). These results suggest that PR55γ is a regulator of the JNK signaling pathway when activated by diverse stimuli.

**Figure 3:**
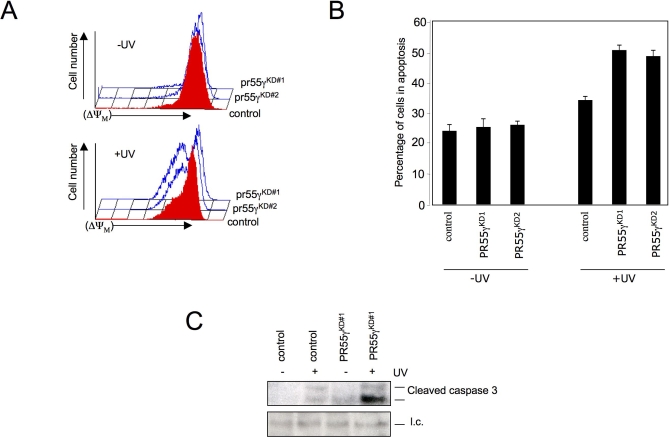
PR55γ Is a Regulator of Apoptosis following UV Irradiation (A) U2-OS cells expressing PR55γ shRNAs as indicated. Selected cells were exposed to UV irradiation (50 J/m^2^) and treated 18 h later with fluorescent dye measuring mitochondria membrane potential (DiOC6[3)]. FACS scan analysis of treated cells is shown. Right and left peaks reflect living and apoptotic cells, respectively. Top panel graph represents breakdown of the mitochondrial membrane potential in the absence of UV. Bottom panel graph represents breakdown of the mitochondrial membrane potential in the presence of UV. (B) Shown is the percentage of apoptotic cells treated with or without UV. Figure represents three independent experiments. (C) U2-OS cells were cotransfected with PR55γ^KD^ vector #2 or control vector. Selected cells were exposed to UV irradiation (50 J/m^2^) and incubated for a further 3 h. Protein samples were analyzed by immunoblotting with antibodies targeting phosphorylated cleaved caspase 3. A background band denotes loading control. doi:10.1371/journal.pgen.0030218.g003

It has previously been established that activation of JNK by UV irradiation can enhance apoptosis in cell culture [30]. Since knockdown of PR55γ leads to enhanced JNK activation, we asked whether knockdown of PR55γ could enhance apoptosis following UV irradiation. UV-induced apoptosis was indeed significantly enhanced in PR55γ-depleted cells (Figure 3A) as determined by measuring the mitochondrial membrane potential with a fluorescent dye (3,3′-dihexyloxa-carbocyanine iodide, [DiOC6 (3)]). Figure 3B represents three independent DiOC6 experiments demonstrating the percentage of apoptosis with or without UV in presence of knockdown vectors targeting PR55γ or a control vector. We also observed an increase in caspase 3 cleavage, a primary executioner of apoptosis, in lysates of cells exposed to UV irradiation, when PR55γ was suppressed (Figure 3C). Similar results were obtained with a second shRNA targeting PR55γ (unpublished data).

### Inhibition of JNK Activity by PR55γ Is Dependent on c-SRC

**Figure 4:**
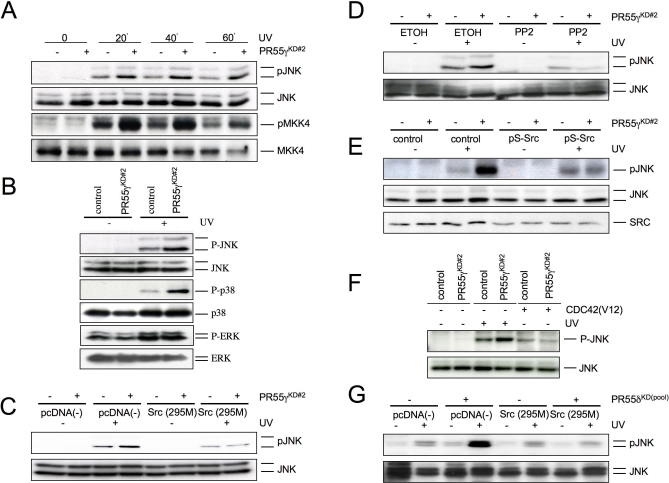
PR55γ Regulates JNK Upstream of MKK4 and at the Level or Upstream of c-SRC (A) pJNK and pMKK4 in relation to the total level of unphosphorylated protein in UV irradiated U2-OS cells followed over time (0–60 min) in the presence or absence of PR55γ^KD2^ vector. (B) U2-OS cells expressing PR55γ^KD2^ or a control vector were treated with UV and incubated for 60 min. Whole cell extracts were probed with the indicated antibodies. (C) PR55γ^KD2^ vector or control vector were cotransfected as indicated in the presence or absence of c-SRC^295M^ (dominant negative). Selected cells were exposed to UV irradiation and whole-cell extracts were analyzed by immunoblotting with antibodies targeting phosphorylated JNK (α-pJNK) or JNK1 and JNK2 (α-JNK). (D) U2-OS cells were cotransfected with PR55γ^KD2^ vector or control vector as indicated and incubated with PP2 for 2 h and UV for 1 h. Whole cell extracts were analyzed by immunoblotting with antibodies targeting phosphorylated JNK (α- pJNK) or JNK1 and JNK2 (α-JNK). (E) PR55γ^KD2^ vector or control vector were cotransfected as indicated in the presence or absence of pSuper-c-SRC. Selected cells were exposed to UV irradiation and whole cell extracts were analyzed by immunoblotting with antibodies targeting phosphorylated JNK (α- pJNK), JNK1 and JNK2 (α-JNK), or SRC(α- SRC). (F) PR55γ^KD2^ vector or control vector were cotransfected as indicated in the presence or absence of CDC42^V12^ (dominant active). Selected cells were exposed to UV irradiation and whole cell extracts were analyzed by immunoblotting with antibodies targeting phosphorylated JNK (α-pJNK) or JNK1 (α-JNK). (G) Cells were transfected with either the PP2A pool targeting PR55δ vector or control vector and cotransfected as indicated in the presence or absence of c-SRC^295M^ (dominant negative). Selected cells were exposed to UV irradiation and whole cell extracts were analyzed by immunoblotting with antibodies targeting phosphorylated JNK (α-pJNK) or JNK1 and JNK2 (α-JNK). doi:10.1371/journal.pgen.0030218.g004

To investigate whether PR55γ regulates the JNK pathway upstream of JNK we asked if loss of PR55γ affected MKK4, the kinase acting directly upstream JNK [31]. We indeed found also that MKK4 activity was significantly enhanced in cells with depleted PR55γ (Figure 4A). These data suggest that PR55γ does not directly affect JNK phosphorylation levels. We therefore asked whether the suppression of PR55γ had an effect on the other MAPK pathways, p38 and ERK. Indeed, western blot analyses indicated that knockdown of PR55γ resulted in increased phosphorylation of both JNK and p38, but not of ERK following UV irradiation (Figure 4B). Thus indicating that PR55γ acts on a key regulatory protein required for activation of both JNK and p38. Of note, no direct interaction was found between PR55γ and components of the MAPK and JNK kinase pathways including the previously described PP2A interacting proteins JNK, MKK4, p38, or c-RAF as determined by coimmunoprecipitation assays (unpublished data) [32–35].

One of the major contributors to the activation of the JNK pathway is the nonreceptor tyrosine kinase c-SRC [12–14]. It was previously shown that the polyoma middle t (MT) antigen, which binds to c-SRC and has been suggested to compete with the PP2A regulatory B subunit for binding to the holoenzyme complex [36–38], is also a potent activator of the JNK signaling cascade [38]. It has recently been described that of the ubiquitously expressed SRC family members only c-SRC [39] and LYN [40] play decisive roles in UV-induced JNK activation. Consistent with this, only c-SRC and Lyn have putative PKC sites in the N-terminal region. However, LYN appears to exclusively regulate JNK kinase but not p38 or ERK. Since knockdown of PR55γ in our system regulates not only JNK but also the MAPK p38 (Figure 4B), it would suggest that c-SRC may be the critical target of PR55γ in negatively regulating the JNK pathway in U2-OS cells following stress.

To test whether the enhanced activation of JNK after suppression of PR55γ is dependent on c-SRC, we cotransfected a dominant negative version of c-SRC, which has a lysine 295 to methionine mutation, resulting in a kinase deficient c-SRC (Src^295M^) [41]. UV irradiation-induced JNK phosphorylation was attenuated in the presence of Src^295M^, in agreement with the earlier finding showing that JNK is activated by both c-SRC independent and c-SRC dependent pathways [39]. However, the enhancing effect of PR55γ knockdown was completely abolished upon coexpression of Src^295M^ (Figure 4C). Likewise inhibition of c-SRC by the generic Src family inhibitor PP2 also inhibited the enhanced JNK activity caused by suppression of PR55γ (Figure 4D). Similarly, cotransfection of a hairpin targeting PR55γ with an shRNA targeting c-SRC completely abolished the enhancing effects of PR55γ on JNK activity (Figure 4E).

To further investigate whether PR55γ can influence the levels of phosphorylated JNK by a non c-SRC family kinase–associated stimulus, we cotransfected a constitutively active form of the GTPaseCdc42 (Cdc42^V12^), which functions upstream of MKK4 in the JNK pathway, in the presence or absence of PR55γ. As expected, transfection of Cdc42^V12^ resulted in activation of JNK [42]. However, the knockdown of PR55γ had no significant affect on JNK activation, whereas it did enhance phosphorylation of JNK following exposure to UV, which served as a control (Figure 4F). Since suppression of a number of the B subunits appeared to enhance the levels of phosphorylated JNK following UV (Figure 1C), we wanted to determine whether the increased levels of phosphorylated JNK observed with knockdown of the other B subunits are dependent on c-SRC. As expected knockdown of PR55δ enhanced the activity of JNK following UV irradiation. Furthermore, like PR55γ, the increased JNK activity was completely attenuated upon cotransfection with kinase dead Src^295M^ (Figure 4G). Together these results suggest that PR55γ and PR55δ negatively regulate JNK signaling in a c-SRC-dependent manner. Since PR55γ is primarily expressed in neuronal tissues and PR55δ is more ubiquitously expressed, it may be that PR55γ and PR55δ mediate the same biochemical responses to stress in different tissues.

### PR55γ Interacts with c-SRC

**Figure 5:**
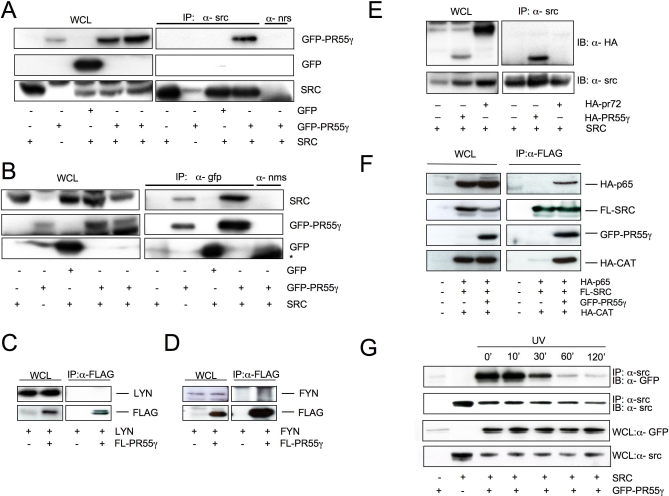
SRC Coimmunoprecipitates with PR55γ (A) U2-OS cells were transfected as indicated. At 48 h after transfection cells were lysed in ELB and c-SRC was immunoprecipitated using a polyclonal rabbit c-SRC antibody or normal rabbit serum (nrs). Coimmunoprecipitated GFP-tagged PR55γ was detected with a GFP antibody. Whole cell lysates (WCL) were probed using c-SRC or GFP antibodies to detect transfected protein. (B) Reverse immunoprecipitation as in (A) GFP was immunoprecipitated using a mouse GFP antibody or normal mouse serum (nms). Coimmunoprecipitated c-SRC was detected with a c-SRC specific antibody. The blot was subsequently reprobed with a GFP antibody to visualize the amount of immunoprecipitated GFP-tagged PR55γ protein. Whole cell lysates (WCL) were probed using c-SRC or GFP antibodies to detect transfected protein. (*) represents the light chain antibody. (C) U2-OS cells were transfected as indicated. Lysates were immunoprecipitated using a FLAG antibody. The blot was probed with a LYN specific antibody and then subsequently reprobed with a FLAG antibody to visualize the amount of immunoprecipitated FLAG-tagged PR55γ protein. (D) U2-OS cells were transfected as indicated. Lysates were immunoprecipitated using a FLAG antibody. The blot was probed with a FYN specific antibody and then subsequently reprobed with a FLAG antibody to visualize the amount of immunoprecipitated FLAG-tagged PR55γ protein. (E) U2-OS were transfected as indicated. c-SRC was immunoprecipitated using a c-SRC antibody. Coimmunoprecipitated HA-tagged PR55γ or HA-tagged PR72 was detected with a HA-specific antibody. The blot was subsequently reprobed with a c-SRC antibody to visualize the amount of immunoprecipitated c-SRC protein. Whole cell lysates (WCL) were probed using c-SRC or HA antibodies to detect transfected protein. (F) Immune precipitation of FLAG-tagged c-SRC and the PP2Ac/PR65 core dimer in HEK 293 cells in the presence (right) or absence (left) of GFP-PR55γ. The left panel shows 10% of the total lysate. (G) U2-OS cells were transfected as indicated. At 48 h after transfection cells were treated with UV (100 J/ m^2^) and incubated for a further 10, 30, 60, and 120 min, respectively. Equal amounts of cell lysate (500 μg) were immunoprecipitated with α- c-SRC antibody. Coimmunoprecipitated GFP-tagged PR55γ was detected with a GFP specific antibody. The blot was subsequently reprobed with a c-SRC antibody to visualize the amount of immunoprecipitated c-SRC protein. Whole cell lysates (WCL) were probed using c-SRC or GFP antibodies to detect transfected protein. doi:10.1371/journal.pgen.0030218.g005

Several studies have suggested a role for PP2A in the regulation of c-SRC [43–45]. For instance, both polyoma MT antigen and adenovirus E4orf4 were previously shown to interact with both c-SRC and PR55α independently, but the relevance of these interactions remained elusive [46–48]. To further address the functional relationship between PR55 and c-SRC, we asked whether PR55γ could physically interact with c-SRC. To investigate this we performed coimmunoprecipitation experiments. We found that immunoprecipitation of c-SRC from lysates of cotransfected cells resulted in coprecipitation of PR55γ (Figure 5A). We also detected this interaction reciprocally by immunoprecipitating GFP-tagged PR55γ with a GFP antibody and then probing the blotted precipitate with a c-SRC antibody (Figure 5B). Importantly, endogenous c-SRC also coimmunoprecipitated with GFP-PR55γ (Figure 5B). Together, these data suggest that PR55γ and c-SRC can form a complex in vivo.

Since PR55γ binds to c-SRC we asked if PR55γ could form physical complexes with other SRC family members. We cotransfected FLAG-PR55γ with either the c-SRC family kinases LYN or FYN. We found that LYN and FYN do not share the ability of c-SRC to interact with PR55γ in coimmunoprecipitation assays (Figure 5C and 5D)

To test if the PR55γ/c-SRC interaction was specific for the B subunit PR55γ we cotransfected c-SRC with PR55γ or the PP2A B^''^ subunit PR72. As shown in Figure 5E, c-SRC physically associated with PR55γ but failed to coimmunoprecipitate with PR72.

Moreover, the specific binding of c-SRC to the B subunit PR55γ suggests that PR55γ is able to recruit the holoenzyme complex to c-SRC. We therefore asked if PR55γ could mediate binding of the PR65/PP2Ac core dimer to c-SRC. We transfected HEK293 cells with constructs expressing FLAG-SRC, HA-PR65, and HA-PP2Ac in the presence or absence of GFP- PR55γ and performed coimmunoprecipitation assays for FLAG-SRC. We found that c-SRC formed a complex with the PP2A holoenzyme exclusively in the presence of PR55γ, indicating that PR55γ is required as bridging factor between c-SRC and the PR65/PP2Ac core dimer (Figure 5F). These observations demonstrate that PR55γ specifically interacts with c-SRC and mediates the recruitment of the PR65/PP2Ac core dimer to c-SRC.

The physical interaction between PR55γ and c-SRC suggests a role as a modulator of c-SRC activity. Since c-SRC activity is increased following UV irradiation, we asked whether UV irradiation could affect the interaction between PR55γ and c-SRC. We followed the interaction between PR55γ and c-SRC after UV irradiation by performing immunoprecipitation experiments. We found that the interaction between PR55γ and c-SRC was gradually lost over time (Figure 5G) demonstrating that the interaction between c-SRC and PR55γ is sensitive to UV irradiation.

### PR55γ Modulates c-SRC Activation

**Figure 6:**
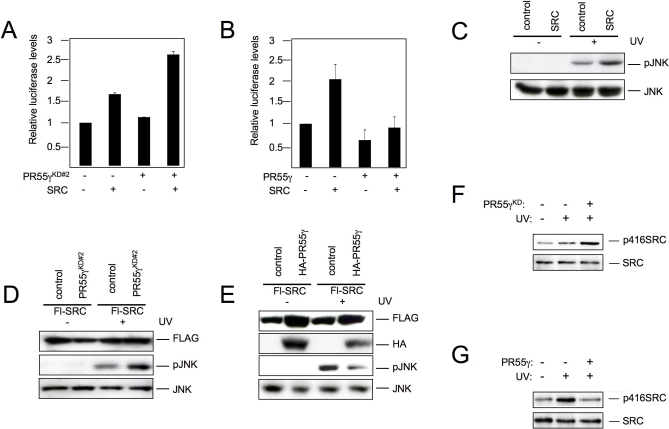
PR55γ Regulates c-SRC-Induced JNK Activation (A) U2-OS cells expressing a 5× AP1-luciferase construct (pGL2), pSUPER-PR55γ, or pcDNA-c-SRC as indicated. Luciferase counts are shown relative to the control. (B) U2-OS cells expressing a 5× AP1-luciferase construct (pGL2), pcDNA-HA-PR55γ, or pcDNA-c-SRC as indicated. Luciferase counts are shown relative to the control. (C) U2-OS cells were transfected with FLAG-SRC or a control vector and treated with UV. Whole cell extracts were probed with the indicated antibodies. (D) U2-OS cells were cotransfected with FLAG-SRC, pSUPER-PR55γ, or a control vector and treated with UV. Whole cell extracts were probed with the indicated antibodies. (E) U2-OS cells were cotransfected with FLAG-SRC, pcDNA-HA-PR55γ, or a empty vector and treated with UV. Whole cell extracts were probed with the indicated antibodies. (F) U2-OS cells expressing pcDNA-HA-hairpins targeting PR55γ or a control vector, and pcDNA-c-SRC were serum starved for 48 h and treated with UV irradiation for 30 min. Cells were lysed in ELB and equivalent amounts of protein were immunoprecipitated with a c-SRC specific antibody. c-SRC phosphorylation was detected with an antibody targeting phosphorylated tyrosine 416 (α-pTyr^416^) and immunoprecipitated c-SRC was detected with an α-c-SRC antibody. (G) U2-OS cells expressing pcDNA-HA-PR55γ or a control vector and pcDNA-c-SRC were serum starved for 48 h and treated with UV irradiation for 30 min. Cells were lysed in ELB and equivalent amounts of protein were immunoprecipitated with a c-SRC specific antibody. c-SRC phosphorylation was detected with an antibody targeting phosphorylated tyrosine 416 (α-pTyr^416^) and immunoprecipitated c-SRC was detected with an α-c-SRC antibody. doi:10.1371/journal.pgen.0030218.g006

Since PR55γ appears to regulate JNK activation at the level of c-SRC, we examined the role of PR55γ on c-SRC- activated transcription of a JNK responsive luciferase reporter. We found that suppression of PR55γ enhanced the ability of c-SRC to activate this reporter (Figure 6A). Conversely, overexpression of PR55γ represses the ability of c-SRC to activate this reporter (Figure 6B). Consistent with these results, western blot analyses demonstrate that overexpression of c-SRC causes an increase in JNK phosphorylation after UV (Figure 6C). Moreover, when we cotransfected short hairpins targeting PR55γ in the presence of c-SRC, we observed that suppression PR55γ enhanced the levels of phosphorylated JNK compared to c-SRC alone (Figure 6D). Consistent with this, ectopic expression of PR55γ inhibited the synergistic activation of JNK mediated by c-SRC and UV (Figure 6E). These results demonstrate that PR55γ is able to influence c-SRC-mediated signaling to the JNK pathway.

To assess whether PR55γ directly modulates c-SRC kinase activity, we evaluated c-SRC Tyr^416^ phosphorylation, a hallmark of its activity [2], by western blot analyses. We found that knockdown of PR55γ could further enhance c-SRC Tyr^416^ phosphorylation following stimulation by UV irradiation (Figure 6F). In contrast overexpression of PR55γ could reduce c-SRC Tyr^416^ phosphorylation following stimulation by UV irradiation (Figure 6G). Together, these results suggest that PR55γ is an inhibitor of c-SRC activity.

### PR55γ Mediates Dephosphorylation of Serine 12 on c-SRC

**Figure 7:**
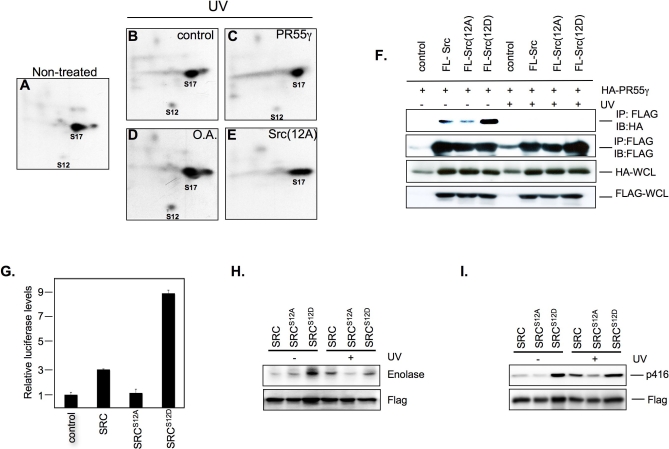
PR55γ Regulates Serine 12 Phosphorylation on c-SRC (A) Phosphotryptic pattern of U2-OS cells expressing c-SRC from untreated cells. (B–E) Phosphopeptides of c-SRC following treatment with UV (100 J/ m^2^) for 30 min in the presence of 2 mCi [^32^P]orthophosphate (B), c-SRC plus PR55γ (C), c-SRC pretreated for 1 h with 1 μM okadaic acid (D), and Ser12A mutant (E). Phosphorylated c-SRC was immunoprecipitated, and samples were subjected to phosphopeptide mapping as described under “Materials and Methods”. (F) U2-OS cells were transfected as indicated. 48 h after transfection cells were treated with UV (100 J/m^2^) and incubated for a further 120 min. Cells were lysed in ELB, and equal amounts of cell lysate (500 μg) were immunoprecipitated using a FLAG antibody (Sigma). Coimmunoprecipitated HA-tagged PR55γ was detected with an HA antibody. Whole cell lysates (WCL) were probed using FLAG or HA antibodies to detect transfected protein. (G) U2-OS cells expressing a 5× AP1-luciferase construct (pGL2), Flag-SRC, Flag- SRC^S12A^, Flag- SRC^S12D^, or control vector as indicated. Luciferase counts are shown relative to the control. (H) U2-OS cells were transfected as indicated. At 48 h after transfection cells were washed twice in PBS and incubated for a further 72 h in medium without FCS. Cells were treated with UV (200 J/m^2^) and incubated for a further 30 min in the same medium. Equal amounts of cell lysate (500 μg) were immunoprecipitated with α-Flag antibody and in vitro kinase assays were performed using enolase as a substrate. (I) U2-OS cells were transfected as indicated. At 48 h after transfection cells were washed twice in PBS and incubated for a further 72 h in medium without FCS. Cells were treated with UV (200 J/m^2^) and incubated for a further 30 min in the same medium. Equal amounts of cell lysate (500 μg) were immunoprecipitated with α-Flag antibody and analyzed by western blot for either tyrosine phosphorylation at residue 416 (top panel) or FLAG (bottom panel). doi:10.1371/journal.pgen.0030218.g007

Several studies have indicated that pretreatment with the PP2A inhibitor okadaic acid (O.A.) induces the phosphorylation of the PKC phosphorylation site, Ser12 on c-SRC, while simultaneously stimulating c-SRC kinase activity [45,49]. To further investigate if PR55γ alters the phosphorylation status of SRC^Ser12^, we stimulated U2-OS cells with UV irradiation in the presence of [^32^P] orthophosphate and performed a 2D tryptic phospho-peptide analysis of phosphorylated c-SRC. Indeed, comparisons with tryptic phosphopeptide maps indicate that overexpression of PR55γ decreased the levels of the phosphorylated Ser12 peptide while okadaic acid slightly increased the phosphorylation levels of the Ser12 peptide, when compared to control samples (Figure 7A–7E). Of note peptide maps showed similar patterns to those performed by Moyers et al. [47]. Similar results were observed when the cells were treated with phorbal 12-myristate 13-acetate (PMA) and Forskolin, potent activators of PKC and PKA, respectively (unpublished data). From these data we conclude that treatment with either UV or PMA induces the phosphorylation of the PKC site Ser12 on c-SRC and that this specific phosphorylation event is significantly diminished in cells overexpressing PR55γ. Of note, no direct interaction was observed between PR55γ and PKCδ, the kinase that directly phosphorylates Ser12 of c-SRC, as determined by coimmunoprecipitation assays (unpublished data).

Since phosphorylation of a specific target sequence has been suggested to be one of the requirements for the targeting of the B regulatory subunit and the subsequent recruitment of the PP2A holoenzyme to the substrate, we wanted to address whether recruitment of PR55γ to c-SRC is dependent upon the phosphorylation status of residue Ser12. To answer this question we generated a gain-of-function (SRC^S12D^) and a loss-of-function (SRC^S12A^) mutant of this phospho-site. To test if phosphorylation of Ser12 was required for the binding of PR55γ we cotransfected HA-PR55γ with either: FLAG-SRC, FLAG- SRC^S12D^, or FLAG- SRC^S12A^ in the presence or absence of UV irradiation and performed coimmunoprecipitation assays. As shown in Figure 7F, mutation of serine 12 to an alanine decreased the association with PR55γ compared to wild-type c-SRC, while the association of PR55γ with SRC^S12D^ significantly increased. However, the association of PR55γ with either wild-type c-SRC or the mutant forms of SRC^Ser12^ were completely abolished following UV irradiation. Together these results suggest that phosphorylation of Ser12 is one of the factors determining PR55γ affinity towards c-SRC and demonstrates that the interaction between PR55γ and c-SRC is sensitive to UV irradiation regardless of the presence of a phospho-moiety on Ser12.

To determine whether the effects of PR55γ on SRC^Ser12^ phosphorylation were the cause of PR55γ-mediated c-SRC regulation, we examined the role of SRC^Ser12^ on a JNK-responsive luciferase reporter. We found that overexpression of the SRC^S12A^ mutant significantly inhibited c-SRC's ability to activate a JNK-responsive luciferase promoter (Figure 7G). Conversely, overexpression of SRC^S12D^ enhanced c-SRC's ability to activate the JNK-responsive luciferase promoter (Figure 7G).

Since SRC^S12D^ and SRC^S12A^ significantly enhanced and diminished c-SRC's ability to activate the JNK-responsive luciferase promoter respectively, we wanted to determine whether phosphorylation of this site affects c-SRC kinase activity. Kinase activity was assayed by monitoring the levels of phosphorylated enolase as an exogenous substrate. As shown above (Figure 6F and 6G), exposure to UV irradiation increases the activation of c-SRC compared to nonstimulated cells (Figure 7H). However, the c-SRC kinase activity was severely crippled in cells expressing SRC^S12A^. Furthermore, c-Src kinase activity was significantly enhanced in SRC^S12D^ cells compared to controls in unstimulated cells (Figure 7H). Similar results were detected when measuring the autophosphorylation of c-SRC at Tyr^416^ (Figure 7I). Taken together these results demonstrate that phosphorylation of Ser12 on c-Src is one of the requirements for full activation of the protein following stress.

### Inhibition of JNK Activity by PR55γ Is Dependent on c-SRC Ser12

**Figure 8:**
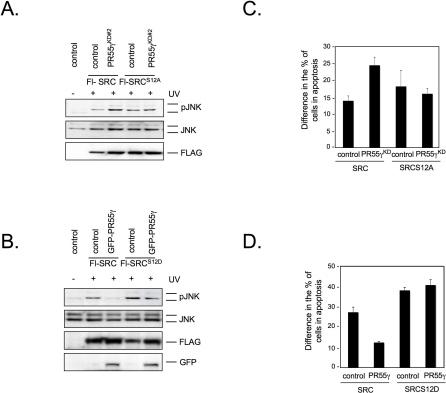
Inhibition of JNK Activity by PR55γ Is Dependent upon Ser12 of c-SRC (A) PR55γ^KD2^ vector or control vector were cotransfected as indicated in the presence of FLAG-SRC or FLAG- SRC^S12A^. Selected cells were exposed to UV irradiation and whole cell extracts were analyzed by immunoblotting with antibodies targeting phosphorylated JNK (α-pJNK), JNK1 and JNK2 (α-JNK), or FLAG (α-FLAG). (B) GFP-PR55γ vector or control vector were cotransfected as indicated in the presence of FLAG-SRC or FLAG- SRC^S12D^. Selected cells were exposed to UV irradiation and whole cell extracts were analyzed by immunoblotting with antibodies targeting phosphorylated JNK (α- pJNK), JNK1 and JNK2 (α-JNK), FLAG (α-FLAG), or GFP (α-GFP). (C) U2-OS cells coexpressing PR55γ shRNAs and FLAG-SRC or FLAG- SRC^S12A^ as indicated. Selected cells were exposed to UV irradiation (50 J/m^2^) and treated 18 h later with fluorescent dye measuring mitochondria membrane potential (DiOC6[3]). Figure represents three independent experiments. (D) U2-OS cells expressing PR55γ and FLAG-SRC or FLAG- SRC^S12D^ as indicated. Selected cells were exposed to UV irradiation (50 J/m^2^) and treated 18 h later with fluorescent dye measuring mitochondria membrane potential (DiOC6[3]). Figure represents three independent experiments. doi:10.1371/journal.pgen.0030218.g008

To determine if the observed effect on JNK activity by PR55γ following UV stimulation is dependent on Ser12, we cotransfected hairpins targeting PR55γ with wild-type c-SRC or the SRC^S12A^ mutant and measured the levels of phosphorylated JNK by western blotting following UV irradiation. As expected knockdown of PR55γ intensified the effect on JNK phosphorylation compared to c-SRC alone. However, the enhancing effect of PR55γ knockdown on JNK phosphorylation was completely attenuated upon coexpression on the SRC^S12A^ mutant (Figure 8A). In agreement with this result, cotransfection of SRC^S12D^ interfered with PR55γ's ability to inhibit JNK phosphorylation following exposure to UV (Figure 8B). Our data collectively demonstrate that modulation of SRC^S12^ phosphorylation by PR55γ is critical for PR55γ's effects on JNK activation.

We next asked whether SRC^S12A^ could abrogate the enhanced apoptosis observed with knockdown of PR55γ. We cotransfected hairpins targeting PR55γ with either SRC^S12A^ or wild-type c-SRC and quantified apoptosis using DiOC6 staining following exposure to UV irradiation. In line with results above (Figure 3A), knockdown of PR55γ increased UV-induced apoptosis in the presence of wild-type c-SRC. However, coexpression of SRC^S12A^ completely curtailed the enhancing effect of PR55γ suppression (Figure 8C). In contrast, coexpression of PR55γ and c-SRC repressed UV-induced apoptosis compared to c-SRC alone, while these effects were completely abolished when PR55γ was coexpressed with SRC^S12D^ (Figure 8D). Taken together, these results demonstrate that the regulation of c-SRC by PR55γ and its subsequent effects on cell survival are mediated through regulation of Ser12 phosphorylation on c-SRC.

## Discussion

It has previously been proposed that the stress response to environmental stimuli mediated by the JNK pathway is regulated by PP2A protein phosphatase activity [33,50,51], although the way in which this pathway is regulated and the specific PP2A holoenzyme responsible for this regulation have not been identified. Via an RNAi-mediated gene family–knockdown screen of regulatory PP2A B subunits, we now identify PR55γ as a specific negative regulator of stress-induced JNK activation. We find that PR55γ regulates the JNK pathway through negative regulation of c-SRC kinase activity. Importantly, we identify here c-SRC serine 12 as a critical residue for the regulation of the c-SRC kinase activity during stress signaling. We show that PR55γ physically interacts with c-SRC and has a higher affinity for c-SRC when it is phosphorylated on serine 12. Since the interaction of the PP2A holoenzyme complex with c-SRC is dependent on PR55γ, this would suggest a transient interaction between PR55γ-containing PP2A holoenzyme and c-SRC, which is reduced as soon as serine 12 dephosphorylation has occurred.

Previous work has indicated that PP2A might play a role in the regulation of c-SRC activity, since treatment of cells with okadaic acid, a chemical inhibitor of PP2A [22], resulted in enhanced c-SRC activity [45], and PP2A can inactivate c-SRC in vitro [43]. Interestingly, polyoma MT is able to compete with the PP2A B regulatory subunit for interaction with the PR65/PP2Ac core dimer [38], and overexpression of polyoma MT is able to activate c-Jun kinase by virtue of its interaction with PP2A [29]. Furthermore, polyoma MT was also found to interact with c-SRC [36] leading to its activation [37]. Moreover, it was reported that adenovirus E4orf4 can interact with both c-SRC and PR55α independently and that the interaction with c-SRC is required for E4orf4 to induce apoptosis [46,52]. Overexpression of E4orf4 phenocopies loss of PR55 in yeast [53], allowing the possibility that inhibition of PR55α is a prerequisite for E4orf4-induced apoptosis in mammalian cells. Our data identifying PR55γ as a negative regulator of c-SRC are in agreement with these studies and could suggest that these viral proteins may function to displace PR55γ from c-SRC.

It has previously been reported that JNK is activated by both c-SRC independent and c-SRC dependent pathways [39]. This present study confirms and extends these results by demonstrating that the inhibition of SRC by PR55γ does not completely inhibit JNK activation but rather results in an overall decrease, similar to the effects observed with a kinase dead mutant of c-SRC. In contrast, knockdown of PR55γ increases SRC kinase activity following UV resulting in enhanced levels of phosphorylated JNK. These results suggest that modulation of one of the upstream activator pathways may result in a prolonged and amplified JNK effect.

It has previously been shown that the majority of c-SRC is present in the perinuclear region where it was found to be inactive as judged by Tyr 416 phosphorylation [54]. c-SRC was also found in the cytoplasm at lower levels correlating with increasing activity and moved to the membrane in response to various stimuli where it was fully active [54]. Similarly we found PR55γ to be primarily expressed in the perinuclear region indicating that PR55γ may colocalise with c-SRC (unpublished data). These data suggest that PR55γ may interact with c-SRC within the perinuclear region inhibiting the induction of c-SRC by PKC by limiting the phosphorylation status of Ser12. Since PR55γ did not decrease the overall levels of other phosphorylation sites within the unique region of c-SRC primarily SER17, phosphorylation of which has been shown to be involved in SRC dependent ERK signaling [55], it suggests that the selective response of c-SRC following PKC phosphorylation at Ser12 may reflect the restricted activation of the JNK downstream effector pathway through either, phosphorylation dependent changes in subcellular localization, as suggested by Liebenhoff et al. who demonstrated that cytoskeletal association of pp60c-src is dependent on phosphorylation of pp60c-src at Ser12 by PKC [56] or by regulation of the binding of proteins that may function to regulate the activity of c-SRC towards JNK.

**Figure 9:**
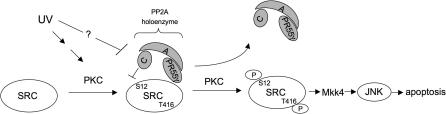
Model for the Regulation of c-SRC-Induced JNK Activation following UV Signaling by PP2A Complexes doi:10.1371/journal.pgen.0030218.g009

One of the intriguing findings of this study is that upon treatment of cells with UV irradiation the interaction between c-SRC and PR55γ is lost. We propose a model in which we suggest that in response to stress c-SRC is activated in part by losing the interaction with its inhibitor allowing c-SRC to be localized to the plasma membrane and subsequent activation of the downstream JNK effector pathways (Figure 9). Similarly, it was described for PR55α that its interaction with the PP2Ac/PR65 dimer is sensitive to gamma irradiation [57]. Further work will be required to reveal the mechanism of UV-induced dissociation of the c-SRC/PR55γ in response to stress.

## Materials and Methods

### Plasmids and antibodies.

pcDNA3- FLAG-SRC, pEGFP-SRC, pMT-SRC, pMT-SRC(527), pMT-SRC(295), pcDNA-LYN, and pcDNA-FYN (Table S2) were kindly provided by P. Stork, G. Superti-Furga, W. Molenaar, and J. Borst. All other Flag-, GFP-, and HA-coding constructs were generated using pcDNA (Invitrogen). Detailed cloning information will be provided upon request. PP2A knockdown library vectors were generated by annealing the individual oligonucleotide primer pairs and cloning them into pSuper as described in [58].The bacterial colonies of each B subunit were then pooled and used for plasmid preparation. The extra shRNA (E) that gave the most efficient knockdown against PR55γ as described in Figure 2A was generated by ligating synthetic oligos (Sigma) against the target sequence 5′-CATGGAGGCAAGACCCATAG-3′ into pSuper. The c-SRC knockdown sequence was obtained from Gonzalez et al. and cloned into pSuper [59]. Antibodies anti-p-JNK, anti-p-MKK-4, anti-p-Src(416), and cleaved caspase-3 were from Cell Signaling; anti-SRC, anti-JNK (C-17), anti-MKK4, HA (Y11), anti-GFP, and anti-FYN were purchased from Santa Cruz Biotechnology Inc. The anti-LYN antibody was a kind gift from J. Borst.

### Cell culture, transient transfections, and Luciferase assays.

All cells were cultured in Dulbecco's modified Eagle medium (DMEM) supplemented with 10% fetal calf serum, D-Glutamate, and Penicillin/Streptomycin. U2-OS cells were divided in 10-cm dishes 1 d prior to transfection. Subconfluent cells were transfected using the calcium phosphate transfection method [60]. Cells were incubated overnight, washed in PBS, and puromycin selected (1.5 μg/ml) for 48 h. When required cells were serum starved for 48 h prior to stimulation. The cells were not allowed to reach confluency. For the screen and subsequent knockdown experiments, U2-OS cells were cotransfected with 20 μg of pooled PP2A shRNAs and 1 μg of pBabe-puro. After 72 h, selected cells were trypsinized and 5 × 10^5^ cells were plated out in a 10-cm dish. After incubating overnight cells were exposed to UV irradiation (100 J/m^2^) and incubated for a further 60 min in the same medium. The following agents were used to stimulate cells: 50 ng/ml EGF (Upstate), 10 ng/ml TNF (Sigma), 10 ng/ml Insulin (Sigma), 500 mM NACL, or UVC (254 nm, 100 J/m^2^). Luciferase assays were performed using the Dual luciferase system (Promega). AP1 luciferase vector (300 ng) was transfected in the presence of CMV-c-SRC (0.5 μg) or a control vector and CMV-Renilla (0.25 μg). For loss of function, 2.5 μg of pSuper vector [58] was cotransfected, and luciferase counts were measured 72 h post-transfection using a TD-20/20 Luminocounter (Promega). For gain-of-function assays, 0.5 μg of CMV construct or control vector (empty CMV) was cotransfected, and luciferase counts were measured 48 h post-transfection.

### Apoptosis assay.

For detection of apoptotic cells, selected cells were incubated for 72 h, trypsinized, and incubated for another 10–12 h in new media. The cells were washed twice in PBS and incubated for 18–24 h following UV treatment (50 J/m^2^), trypsinized, washed once with PBS, and resuspended for 10–15 minutes in 250 μl PBS containing 40 nM DiOC6 (3). After incubation the cells were analyzed by FACS analysis.

### Western blotting and coimmunoprecipitation.

Cells were lysed in solubilizing buffer (50 mM Tris [pH 8.0], 150 mM NaCl, 1 % NP-40, 0.5% deoxycholic acid, 0.1% SDS, 1 mM Sodium Vanadate, 1 mM pyrophosphate, 50 mM sodium fluoride, 100 mM β-glycerol phosphate), supplemented with protease inhibitors (Complete; Roche). Whole cell extracts were then separated on 7%–12% SDS-Page gels and transferred to polyvinylidene difluoride membranes (Millipore). Membranes were blocked with bovine serum albumin and probed with specific antibodies. Blots were then incubated with an HRP-linked second antibody and resolved with chemiluminescence (Pierce). For coimmunoprecipitations, cells were lysed in ELB (0.25 M NaCl, 0.1%NP-40, 50 mM HEPES [pH 7.3]) supplemented with protease inhibitors. Lysates were then incubated for 2 h with 2 μg of the indicated antibodies conjugated to protein A or protein G sepharose beads, washed three times in ELB buffer, and separated on SDS-PAGE gels. When appropriate cell lystates were immunoprecipitated with ANTI-FLAG M2 Affinity Gel (Sigma).

### 2D tryptic phospho-peptide analysis and phospho-labeling.

For tryptic phosphopeptide analysis U2-OS cells were cotransfected with 4 μg Flag-Src or 4μg or Flag-Src (12A) and 20 μg PR55γ or control vector. Cells were phospho-starved for 45 min and 2 mCi of [^32^ P] orthophosphate was then added to the cells and incubated an additional 3 h. PMA at a final concentration of 200 nM was added for 30 min at 37 °C or the cells were treated with UV irradiation (100 J/m^2^) and incubated for a further 30 min at 37 °C. c-SRC was immunoprecipitated with Flag antibody (Sigma) as described above. The entire sample was loaded onto an SDS-PAGE gel, run, and then dried. The film was then exposed for 3 h at room temperature. The radioactive bands were then isolated, proteins eluted, digested with trypsin, and phosphopeptide mapping was performed as described previously [61,62]. For phospholabeling analysis HEK 293 cells were cotransfected with 4 μg Flag-PR55γ, 10 μg HA-PR65, and 10 μg HA-PP2Ac. Cells were phospho-starved for 45 min, UV stimulated as above, and then 2 mCi of [^32^ P] orthophosphate was added to the cells and incubated for a further 2 h in the same medium. c-SRC was immunoprecipitated with Flag antibody (Sigma) and an HA antibody (Y11, Santa Cruz) as described above. The entire sample was loaded onto an SDS-PAGE gel, run, and then dried.

## Supporting Information

Table S1 Gene Names and Primer Sequences (114 KB DOC)

Table S2 Gene Names and GenBank Numbers (26 KB DOC)

## Abbreviations

DiOC6 (3): 3,3′-dihexyloxa-carbocyanine iodide
GFP: green fluorescent protein
JNK: c-Jun NH_2_-terminal kinase
mt: middle T
PP2A: Protein Phosphatase type 2A
SH: Src homology
