# Supplementary material for: A RNA Interference Screen Identifies the Protein Phosphatase 2A Subunit PR55γ as a Stress-Sensitive Inhibitor of c-SRC
Source: PLoS Genet. 2007 Dec 7;3(12):e218. doi: 10.1371/journal.pgen.0030218 (PMC2134945; doi:10.1371/journal.pgen.0030218)
Supplement: Supplementary file 1 [file pgen.0030218.st001.doc]

Supplementary Table 1

| GENE NAME | Genbank code | B SUBUNIT |  | PRIMER SEQUENCES | |  |
| --- | --- | --- | --- | --- | --- | --- |
|  |  |  |  |  |  |  |
| PPP2R2A | NM_002717.2 | PR55 ALPHA |  | CAG GAG ATA AAG GTG GTA G | | |
|  |  |  |  | GAC CAG AAG GGT ATA ACT T | | |
|  |  |  |  | CAT ATT TAT CTG CAG ATG A | | |
|  |  |  |  | GAA CCC ACT TCC TGC TTA G | | |
|  |  |  |  |  |  |  |
| PPP2R2B | NM_181674.1 | PR55 BETA |  | GGG GGG TCG GGT TGT AAT A | | |
|  |  |  |  | GAG GCC AGA AGG CTA CCA T | | |
|  |  |  |  | GGT TAA CTA GGT GGA CAA G | | |
|  |  |  |  | GAT GGC TCC CCC AGC AGA A | | |
|  |  |  |  |  |  |  |
| PPP2R2C | NM_020416.2 | PR55 GAMMA |  | GAC TCC AGC TGA CAT CAT C (A) | | |
|  |  |  |  | GAT CAA CAA GAT CAA GTG G (B) | | |
|  |  |  |  | GGA TAC AAC CTG AAG GAT G (C) | | |
|  |  |  |  | GGC CCG AAG GAT ACA ACC T (D) | | |
|  |  |  |  | CATGGAGGCAAGACCCATAG | (E) |  |
|  |  |  |  |  |  |  |
| PPP2R2D | NM_001003656.1 | PR55 DELTA |  | GAG TCT ATA ATT GGA GAT C | | |
|  |  |  |  | CTA TAG GCT TGC ACT GCC A | | |
|  |  |  |  | TCG GAC ATC ATT TCC ACC G | | |
|  |  |  |  | GAG CAC AGA GCG GAA GTT C | | |
|  |  |  |  |  |  |  |
| PPP2R5A | NM_006243 | PR61 ALPHA |  | GTG TGT ATC CAG TTC TCA T | | |
|  |  |  |  | GGA ATT GGA ACG TGA AGA A | | |
|  |  |  |  | CAC TGA ATG AAC TGG TTG A | | |
|  |  |  |  | GTT GCA GCA GTG TTG TAT A | | |
|  |  |  |  |  |  |  |
| PPP2R5B | NM_006244 | PR61 BETA |  | GCT GCC CCC TGC AAG CAC C | | |
|  |  |  |  | GTT TGT CTT GAT GCT CCT G | | |
|  |  |  |  | CCA CAT CTT CCT CCG GTT C | | |
|  |  |  |  | CGA GCT GGT GGA GTG TGT G | | |
|  |  |  |  |  |  |  |
| PPP2R5D | NM_006245 | PR61 DELTA |  | GCC CAG TCT CAG CCA CCG T | | |
|  |  |  |  | CCT CAG CAA GAA TCG GGA G | | |
|  |  |  |  | GAA GTA CAT CGA CCA GAA G | | |
|  |  |  |  | GTG TGT CTC TAG CCC CCA T | | |
|  |  |  |  |  |  |  |
| PPP2R5E | NM_006246 | PR61 EPSILON |  | GTT TCT TGG TCT TAG AGC T | | |
|  |  |  |  | A CAG TTT CTG GTG AAA GTA | | |
|  |  |  |  | GAA TAC AAG CGC TCC ACT C | | |
|  |  |  |  | CTC TCC CTC CTA GTG ACA G | | |
|  |  |  |  |  |  |  |
| PPP2R5C | NM_178587.1 | PR61 GAMMA 1 |  | GCC AAT CCC CAG GTA CTA A | | |
|  |  |  |  | GAT AGG AAT ATA TGT CTT C | | |
|  |  |  |  | TAT ATG TCT TCA CCA TGG G | | |
|  |  |  |  | CGC CCA GGG TTA CTT GAA T | | |
|  |  |  |  |  |  |  |
| PPP2R5C | NM_178586.1 | PR61 GAMMA 2/3 |  | GGA CCG TCC TCT TGC ACT C | | |
|  |  |  |  | GAT CCG AAG AAG GAC CGT C | | |
|  |  |  |  | GTC CGA GCT GCC TCA GGA C | | |
|  |  |  |  | GCC TTG GAA GCT CAC TGC A | | |
|  |  |  |  |  |  |  |
| PPP2R5C | NM_002719.2 | PR61 GAMMA 3 |  | GCC AGC ACC ATG AGC ATT C | | |
|  |  |  |  | TGG AGA CAG ATG GGC CTT T | | |
|  |  |  |  | GAT GTG CAG ATG CTG AGA A | | |
|  |  |  |  | GAC AGT GAA GGA CGA GGC T | | |
|  |  |  |  |  |  |  |
|  |  |  |  |  |  |  |
| PPP2R3A | NM_181897.1 | PR72 |  | GGG ACC CGG ATT TAA GGG G | | |
|  |  |  |  | GGG GAG AGC TAG CTT TCC T | | |
|  |  |  |  | GCG GCT GAA GTC ATT TCA G | | |
|  |  |  |  | CGG ATG GAA GCC ATG GGA A | | |
|  |  |  |  |  |  |  |
| PPP2R3A | NM_002718.3 | PR130 |  | CGA TGG GAA CGC CCC ATC C | | |
|  |  |  |  | GAT GTG CTT GGA CAT CTT A | | |
|  |  |  |  | CCC CCT AAA TAT GAA GAT G | | |
|  |  |  |  |  |  |  |
| PPP2R3B | NM_013239.3 | PR48 |  | CACGCACCCGGGGCTGTCG | | |
|  |  |  |  | CGTCTTCTTCGACACCTTC | |  |
|  |  |  |  |  |  |  |
|  |  |  |  |  |  |  |
|  |  |  |  | GGC CGA GTG CCT GGA GAA G | | |
| PPP2R4 | NM_021131 | PR53 |  | GTT CCC TGT GAT CCA GCA C | | |
|  |  |  |  | GGC CGT GAA TGA GAA CCA C | | |
|  |  |  |  | GGA GCT AGT GGG GAA CTC C | | |
|  |  |  |  |  |  |  |
| PP2ACAT | NM_004156.2 | CATALYTIC |  | AGT ATG GGA ATG CCA ACG T | | |
|  |  |  |  | ATC ACC GGA TAC AAA CTA C | | |
|  |  |  |  | TGC CAA CGT TTG GAA ATA T | | |
|  |  |  |  | CGC ATT ACA ATA TTG AGA G | | |
|  |  |  |  |  |  |  |
|  |  |  |  |  |  |  |
