# Supplementary material for: A RNA Interference Screen Identifies the Protein Phosphatase 2A Subunit PR55γ as a Stress-Sensitive Inhibitor of c-SRC
Source: PLoS Genet. 2007 Dec 7;3(12):e218. doi: 10.1371/journal.pgen.0030218 (PMC2134945; doi:10.1371/journal.pgen.0030218)
Supplement: Supplementary file 2 [file pgen.0030218.st002.doc]

Supplementary Table 2

| Gene Name | GenBank code |
| --- | --- |
| c-SRC | NP_005408 |
| FYN | P13406 |
| LYN | P07948 |
| JNK | NP_002741 |
| MKK4 | NP_003001 |
| CDC42 | CAI19851 |
